# Variability in prion protein genotypes by spatial unit to inform susceptibility to chronic wasting disease

**DOI:** 10.1080/19336896.2022.2117535

**Published:** 2022-09-14

**Authors:** Alberto F. Fameli, Jessie Edson, Jeremiah E. Banfield, Christopher S. Rosenberry, W. David Walter

**Affiliations:** a Pennsylvania Cooperative Fish and Wildlife Research Unit, The Pennsylvania State University, University Park, PA, USA; b Pennsylvania Game Commission, Bureau of Wildlife Management, 2001 Elmerton Avenue,Harrisburg, PA, USA; c U.S. Geological Survey, Pennsylvania Cooperative Fish and Wildlife Research Unit, 403 Forest Resources Building, The Pennsylvania State University, University Park, PA, USA

**Keywords:** Chronic wasting disease, prion protein gene, elk, genetic variability, spatial variability

## Abstract

Chronic wasting disease (CWD) is a fatal encephalopathy affecting North American cervids. Certain alleles in a host’s prion protein gene are responsible for reduced susceptibility to CWD. We assessed for the first time variability in the prion protein gene of elk (*Cervus canadensis*) present in Pennsylvania, United States of America, a reintroduced population for which CWD cases have never been reported. We sequenced the prion protein gene (PRNP) of 565 elk samples collected over 7 years (2014–2020) and found two polymorphic sites (codon 21 and codon 132). The allele associated with reduced susceptibility to CWD is present in the population, and there was no evidence of deviations from Hardy-Weinberg equilibrium in any of our sampling years (*p*-values between 0.14 and 1), consistent with the lack of selective pressure on the PRNP. The less susceptible genotypes were found in a frequency similar to the ones reported for elk populations in the states of Wyoming and South Dakota before CWD was detected. We calculated the proportion of less susceptible genotypes in each hunt zone in Pennsylvania as a proxy for their vulnerability to the establishment of CWD, and interpolated these results to obtain a surface representing expected proportion of the less susceptible genotypes across the area. Based on this analysis, hunt zones located in the southern part of our study area have a low proportion of less susceptible genotypes, which is discouraging for elk persistence in Pennsylvania given that these hunt zones are adjacent to the deer Disease Management Area 3, where CWD has been present since 2014.

## Introduction

Incorporating genetic data in studies to help promote population persistence generally involves assessing variability at multiple non-coding (i.e. neutral) genetic markers, assuming their correlation to fitness-influencing loci, and implementing actions to retain/increase variability [1–4]. This population-wide genetic variability increases the chances of producing individuals with higher levels of outbreeding, a trait generally displaying a positive relation with fitness. This relationship has been documented, for example, in a higher level of genetic diversity being associated with increased hatching success and fertility in greater prairie chickens (*Tympanuchus cupido pinnatus*) [1], increased yearly recruitment in adders (*Vipera berus*) [5], higher annual reproductive success and survival in big-horn sheep (*Ovis candadensis*) [3], higher lifetime breeding success in red deer (*Cervus elaphus*) [6], higher lifetime copulation success in black grouse (*Tetrao tetrix*) [7], and reduced susceptibility to parasitism by gastrointestinal nematodes in Soay sheep (*Ovis aries*) [8] and capercaillie (*Tetrao urogallus*) [9].

The effect that specific alleles might have on fitness-related traits, however, is usually unknown due to the number of intrinsic and extrinsic factors operating to determine individual fitness [10]. Exceptions to this general observation are the genotypic variants in the prion protein gene (PRNP) which account for reduced susceptibility to chronic wasting disease (CWD), a contagious spongiform encephalopathy that affects North American cervids [11,12]. Aetiology of this fatal disease depends on the conversion of a host’s own prion protein to a misfolded configuration, a process catalysed by an exogenous infectious prion protein [13]. This conversion results in the accumulation of the misfolded form in lymphoid and central nervous system tissues, causing brain lesions responsible for the drastic neurodegenerative process observed [14–16]. The disease continues to spread throughout North America and other parts of the world [17], and several studies have also shown an increase in prevalence in endemic zones [18,19]. Concerns regarding CWD arise from its effect on reducing sustainability of cervid populations and the possibility of spillover to other species (see [17] and [20] for a review). For example, free-ranging white-tailed deer (*Odocoileus virginianus*) that tested positive for CWD were 4.5 times more likely to die annually than non-infected deer [19] and survival rate of infected mule deer (*Odocoileus hemionus*) is half of that expected for non-infected individuals [21]. These articles reporting differences in survival [19,21] also show that demographic models predict population growth rates too low to maintain sustainable populations in the presence of CWD.

Polymorphic sites in PRNP can result in differential susceptibility of cervids to CWD. Certain genotypes show a lower probability of being found CWD positive [22–24] which could be associated with a decreased probability of infection. These genotypes also present a slower progression of the disease [25,26], which might play a significant role in natural populations, as the delay in reaching the prion shedding stage means these animals could die of other causes before becoming infectious [22]. The individuals possessing these genotypes are not completely protected against CWD and can be experimentally infected or acquire the disease in natural conditions [25], but it has been reported that populations characterized by a higher proportion of these genotypes show lower prevalence of CWD [27]. In elk (*Cervus canadensis*), a single nucleotide polymorphism at codon 132 (coding for Met or Leu [11]) is associated with lower susceptibility to the disease, in the form of a longer life expectancy for individuals whose genotype is Met/Leu or Leu/Leu (hereafter ‘less susceptible individuals’) [11,28,29]. A few studies have estimated the proportion of these less susceptible individuals in both native and reintroduced free-ranging elk herds [10,11,30]. This information has not been incorporated into management of free-ranging cervids, despite showing the capacity to greatly influence population sustainability [31].

Elk populations that have not been exposed to CWD show low frequency of the less susceptible genotypes, but detectable shifts in this frequency may occur over a few generations once CWD becomes established in the area [10]. The initial low frequencies of the less susceptible genotypes pose a question regarding the ability of reintroduced herds to cope with CWD upon exposure to it. This concern is motivated by the low number of founders that were used for creating these new herds [32–34], and given the low frequency of the less susceptible genotypes in the source populations, it is unsure whether reintroduction plans managed to represent this variability in PRNP.

Reintroductions of elk in Pennsylvania began following extirpation in the late 1800s, with multiple elk translocations carried out between 1913 and 1926 [32–34]. Demographic history of these new herds was marked by several factors that contributed to a prolonged bottleneck which, coupled with the small number of founders and polygynous mating system, has caused the extremely low genetic variability currently observed in Pennsylvania elk [35]. These factors could also have exacerbated the problem of initial low frequencies of PRNP variants by increasing the effects of genetic drift. Even though there have been no reported cases of CWD-infected elk in Pennsylvania, it has been detected in free-ranging white-tailed deer in the area adjacent to the elk range since 2014 [34], and in other areas of the state since 2012 [36].

Elk hunting in Pennsylvania was closed during 1931 − 2001. Since reopening hunting in 2001, hunt zones in the elk management area have been designed to encompass ‘sub-populations’ (defined as groups of animals that have similar home-ranges throughout the year), and areas where conflict with humans is present or probable (e.g. residential areas) [34]. Proportions of PRNP genotypes have not been documented for these hunt zones or the population as a whole, precluding the use of this information in the design of management plans. Given that assessment of proportions and spatial distribution of PRNP genotypes is warranted, we studied the elk population in Pennsylvania to: 1) estimate these proportions prior to CWD selective pressure and compare them with other native or reintroduced populations, assessing differences in pre-exposure susceptibility and 2) describe the spatial distribution of less susceptible genotypes and their proportion in each hunt zone. This information could potentially assist in focusing disease control efforts in more vulnerable hunt zones, characterized by a low proportion of less susceptible individuals.

## Results

**Figure 1.**
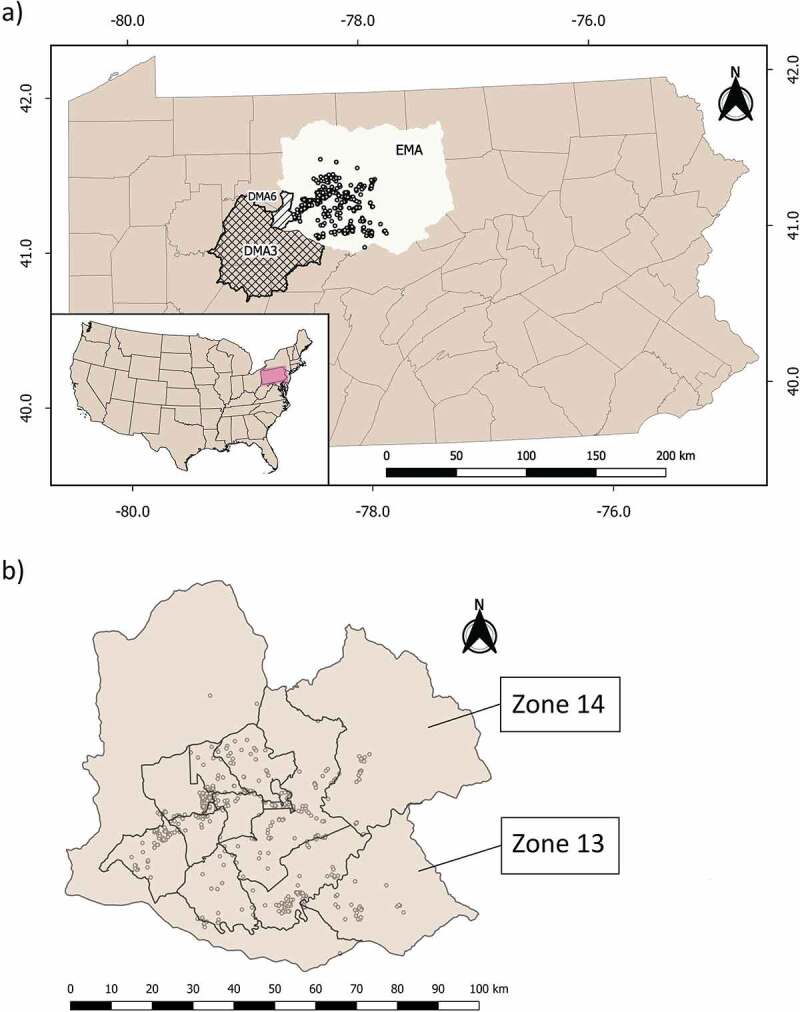
a) Distribution of elk samples (circles) collected in Pennsylvania’s Elk Management Area (EMA, current delineation shown) between 2014 and 2020 (n = 565). Disease Management Area 3 (DMA3, cross-hatched) is adjacent to EMA, while Disease Management Area 6 (DMA6, hatched) is inside EMA. b) Distribution of elk samples (circles) as shown in Figure 2a, and hunt zone delineation used in the period 2018–2020, for which we estimated the proportions of less susceptible genotypes. These hunt zones are located within the Elk Management Area. Two hunt zones were created during our sampling period: zone 13 (incorporated in 2015) and zone 14 (incorporated in 2018).

We obtained 1130 reads corresponding to the forward and reverse PRNP sequences of 565 elk individuals (Figure 1). Seven of these sequences were removed from the analyses due to their low-quality score, but this procedure did not result in a reduction in the number of individuals being analysed (i.e. seven individuals were represented by one sequence instead of two).

We detected two polymorphic sites in our dataset: one at nucleotide 394 (codon 132: atg/ttg) resulting in the amino acid polymorphism Met/Leu, responsible for differential CWD-susceptibility, and the other being a synonymous polymorphism at nucleotide 63 (codon 21: gtc/gtt). All inferred haplotypes had a probability of 100% and showed a high degree of association between genotypes (Table 1) despite the polymorphic sites being separated by 331 bp. In other words, the presence of C at nucleotide 63 almost invariably identified the presence of A at nucleotide 394, and the presence of T at nucleotide 63 was always associated with a T at nucleotide 394. There was no evidence of deviations from the proportions of heterozygous genotypes expected under Hardy-Weinberg equilibrium for codon 132 in any of our sampling years (*p*-values = 0.14 − 1.00).

**Table 1.** Haplotypes inferred based on the two polymorphic sites found in our study of elk from the state of Pennsylvania, U.S.A., and those reported by Chafin et al. [47] for white-tailed deer. For comparison purposes, we only show their results for two polymorphic sites: one (nucleotide 60) in a position similar to the one we found and the other (nucleotide 286) linked to differential CWD-susceptibility in white-tailed deer (similar to nucleotide 394 in elk PRNP sequence).

Elk			White-tailed deer		
Nucleotides 63/394	N	%	Nucleotides 60/286		%
C/A	1016	89.91	C/G		71.46
C/T	1	0.09	C/A		17.24
T/T	113	10	T/G		11.24
T/A	0	0	T/A		0.07

### Comparison of PRNP genotypes

We compared the genotypic frequencies obtained with those reported in five other areas before CWD had been detected [10,11]. Proportions of PRNP genotypes in our study (80.71% Met/Met, 18.41% Met/Leu, 0.88% Leu/Leu) were similar to two other populations,one near Jackson (JN), Wyoming and the other from Black Hills (BH), South Dakota (Table 2, *p*-value= 1.0 for both comparisons), but they differed from populations in Theodore Roosevelt National Park (THRO), North Dakota and Wyoming/Wind River Ranges (WIND), Wyoming. THRO had fewer less susceptible individuals than our sample (*p*-value< 0.001), while these individuals were more abundant in WIND than in our sample (*p*-value= 0.012). No differences were observed between our sample and the population in Absaroka Range (ABSA), Wyoming, *p*-value= 0.27.

**Table 2.** Frequencies of the different PRNP genotypes based on polymorphism at nucleotide 394 in our study, and those reported for other elk populations. N = number of individuals, % = percentage in our sample, JN% = percentage calculated from a sample of 55 individuals from Wyoming [11], BH% = percentage calculated from a sample of 42 individuals from South Dakota [11], WIND% = percentage calculated from a sample of 186 individuals from Wyoming [10], ABSA% = percentage calculated from a sample of 148 individuals from Wyoming [10], THRO% = percentage calculated from a sample of 199 individuals from North Dakota [10], PA% = percentage found in our dataset based on a bootstrapping procedure, adjusting sample size to match the ones obtained in previous studies for different populations.

Pennsylvania			Other populations				
	N	%	JN%	BH%	WIND%	ABSA%	THRO%
			(PA%)	(PA%)	(PA%)*	(PA%)	(PA%)**
Met/Met	456	80.71	80	83	69	74	93
			(80.79)	(80.77)	(80.69)	(80.71)	(80.88)
Met/Leu	104	18.41	20	17	27	24	7
			(18.29)	(18.34)	(18.40)	(18.38)	(18.25)
Leu/Leu	5	0.88	0	0	4	1	1
			(0.91)	(0.89)	(0.90)	(0.91)	(0.87)

*p-value<0.05

**p-value<0.001

### Distribution of less susceptible genotypes

**Figure 2.**
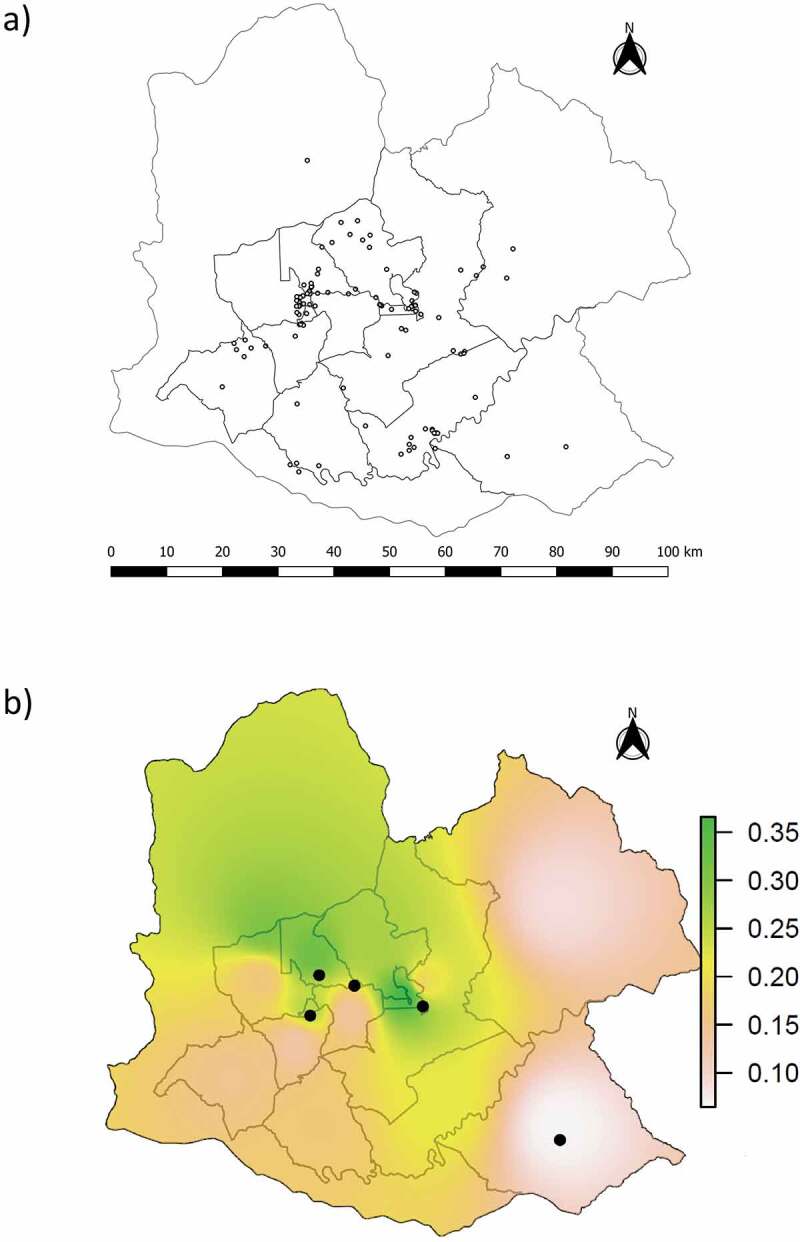
Distribution of less susceptible PRNP genotypes (Met/Leu and Leu/Leu) obtained from our sample of 565 elk, shown as: (a) presence of the 109 less susceptible genotypes using individual GPS coordinates, and (b) interpolation based on the proportion of Met/Leu individuals in each hunt zone, with circles representing Leu/Leu individuals. The interpolation combining both types of less susceptible genotypes showed the same pattern.

Pairwise *F_ST_* comparisons between years did not detect significant differences in allele frequencies (*p*-values = 0.04 − 1.00, Bonferroni corrected α = 0.002), allowing us to combine samples collected from 2014 to 2020. The 109 less susceptible individuals were found throughout the study area, although they seemed to be more concentrated in the central part of this area (Figure 2(a)). To evaluate whether the proportion of less susceptible individuals in hunt zones was consistent over time, we divided our dataset into three periods. We then used Fisher’s exact tests for count data to compare the proportion of less susceptible individuals in the same hunt zone between periods, although this could not be done for all hunt zones due to many of them having less than 15 individuals. None of these inter-period comparisons was significant, giving support to the hypothesis that this proportion was stable over time in each hunt zone. The Clark-Evans coefficient was 0.74, significantly lower than 1 (*p*-value< 0.001) and therefore indicating a clumped distribution of less susceptible individuals. The interpolated frequencies of Met/Leu indicated higher concentrations of this genotype within the central part of our study area (Figure 2(b)) while a low proportion (shown in white and pink) occurs in the north-east, south and south-east. We obtained the same pattern when we mapped the proportion of all less susceptible individuals (Met/Leu and Leu/Leu pooled together, data not shown).

## Discussion

Comparisons between populations before exposure to CWD can be useful in the management of cervids, by enabling evaluation of past management actions carried out in other populations that had similar starting points in terms of PRNP variability (controlling for factors such density, habitat use, predation rate and winter mortality). Current elk management includes creating reliable population models by combining age-specific survival estimates and reproductive data [34], but it does not stipulate the importance of including PRNP genotype proportions in those models. Accounting for differential CWD-mortality of these genotypes has been shown to greatly influence the outcome of such models and has the potential to better predict the future demographic status under various scenarios [37,38].

We are the first to report PRNP genotype frequencies in elk in Pennsylvania, providing a comparison point for the future if CWD becomes established in this population. Despite their severe historical demographic bottleneck and subsequent low genetic variability at microsatellite loci [35], elk in Pennsylvania had similar PRNP genotype frequencies to those found in three other populations before their first confirmed case of CWD (JN, ABSA and BH). In Wyoming, CWD-infected elk had not been detected in the JN population by 1999 or in ABSA by 2008–2015 [10,11]. Prevalence in the area for elk was recently reported, however, to be between >0% and 3% for the period 2017–2021 [39].

Between July 2020 and April 2021, 88 elk samples were tested for CWD in the area near BH with nine of these samples being positive [40], indicating a prevalence of 10%. This increase from 0% [11] to 10% in just over 20 years is alarming given that this population and showed similar PRNP genotype frequencies to our elk sampled in Pennsylvania. Our results differed from the genotype frequencies reported in THRO, where low proportion of less susceptible elk was attributed to translocations from WIND [10]. The authors did not comment on the high proportion of less susceptible individuals found in WIND.

Some researchers have suggested that the prolonged incubation period observed in less susceptible individuals could result in shedding proportionally more infectious prions during their lifetime [13,41], which would be detrimental for the persistence of the population; however, recent studies have found evidence that opposes this hypothesis. For example, it has been reported that cervids with less susceptible genotypes were less likely to excrete prions [42], and areas with higher frequency of less susceptible white-tailed deer have lower CWD prevalence [27]. The latter authors suggested this could be attributed to the less susceptible individuals acting as a barrier that slows down the expansion of CWD.

We found that PRNP genotype frequencies in our elk match what would be expected under Hardy-Weinberg equilibrium for each of our sampling years, which is consistent with a scenario where PRNP is not currently under natural selection. Management actions aimed to prevent CWD transmission could benefit from knowing PRNP genotype proportions in each hunt zone, rather than only knowing these proportions at a population level. In this way, spatial variability in genotype proportions could potentially be used to inform hunt quotas aiming to protect the less susceptible individuals, or focus efforts in more vulnerable zones where the vast majority of individuals are more susceptible to CWD.

In our case it was necessary to pool together samples collected across years, as some hunt zones were represented by only a few individuals in certain years. The less susceptible genotypes were present throughout our study area, but they appeared to be more abundant in the central part of this area when combining all sampling years (Figure 2(a)). This information by itself cannot be used as evidence of low vulnerability to CWD in the central part of the study area because it does not relate this abundance of less susceptible individuals to population size. To assess the relative vulnerability of different hunt zones we calculated the proportions of less susceptible genotypes in each, and interpolated these results to our entire study area. The pattern obtained indicated a higher proportion of less susceptible genotypes in the central region (i.e. lower vulnerability to CWD). This highlights the potential for more focused elk population management in the southern part of the study area, considering that is where proportion of less susceptible individuals is relatively low. In addition, the southern region is in close proximity to Disease Management Area 3 where CWD has been reported in free-ranging white-tailed deer [34]. Considering that populations with similar initial PRNP frequencies showed a rapid increase in prevalence after CWD was established (e.g. ABSA [10]), it is possible that zones closer to Disease Management Area 3 may be more vulnerable to CWD highlighting the importance of our results to future management of elk in Pennsylvania. Previous studies have demonstrated the feasibility of cross-species CWD transmission via oral or intracerebral inoculation, using cervids and transgenic mice expressing cervid prion protein as test subjects [43–45]. The natural determinants and routes of cross-species transmission in the wild are less understood, but its occurrence is considered possible between elk, white-tailed deer and mule deer [46].

Our elk population has been shown to present very low levels of genetic variability [35], which could make it particularly vulnerable to other pathogens/parasites or reduce its breeding success, as it has been reported for other species [3,6]. We found two polymorphic sites, which could potentially be associated in four haplotypes. Three of these haplotypes were present in our dataset, although their frequencies show a high degree of association between the alleles at each polymorphic site separated by 331 bp. This association between polymorphic sites was not as strong in the data reported by Chafin et al. [47] for white-tailed deer, where two polymorphic sites in the PRNP had lower level of association despite being closer to each other in the chromosome (226 bp). Our results of inferred haplotypes are congruent with a scenario of demographic bottleneck, where combinations of alleles found in certain haplotypes were lost due to genetic drift or founder effect, and they have not been recovered by meiotic recombination. Elk management that includes a genetic component that considers the tradeoff between promoting increase in the frequency of less susceptible genotypes and maintaining/increasing genome-wide variability may be warranted.

## Materials and methods

### Study area and sampling

The Pennsylvania elk management area is delineated by the Pennsylvania Game Commission (PGC) and currently extends over 9,731 km^2^ of public and private land [34], composed mainly of mature forest (Figure 1(a)). Regulated harvest as part of the Elk Management Plan is implemented to promote expansion of elk inside the management area and deter their dispersion beyond those borders, where they could generate crop losses and vehicle collisions, or come into contact with CWD-infected deer. Currently, hunt zones are reviewed every 2 years, and their boundaries are defined based on sub-population home ranges that are adjusted to natural and anthropogenic features (e.g., highways, power lines, rivers [34]). The south-west section of the elk management area is adjacent to the third Disease Management Area established in the state, after detection of a captive CWD-positive white-tailed deer in 2014. The sixth Disease Management Area now falls within the western-most quarter of the elk management area as a result of a CWD detection in the third Disease Management Area [48].

We collected samples during the elk hunting season (2014 to 2020), Figure 1(a) that occurs in early November, running for 6 days (Monday–Saturday). Successful hunters must present harvested elk within 24 h of harvest at a mandatory check station operated by the PGC. The annual harvest is approximately 100 elk with the number of hunter licences issued by hunt zone and correlated with elk population in each zone [34]. A tongue sample was collected from each elk by PGC personnel at the elk check station. Additional samples were collected using an ear punch during a study on elk parturition and calf survival in 2020 under the protocol approved by the Institutional Animal Care and Use Committee at The Pennsylvania State University (No. PROTO201901185) and are within the guidelines of the American Society of Mammalogists [49]. We sequenced a subset (n = 565) of the samples collected to achieve a more homogeneous representation of the study area (n_2014_ = 87, n_2015_ = 81, n_2016_ = 87, n_2017_ = 103, n_2018_ = 91, n_2019_ = 55, n_2020_ = 61).

### Sequence analysis

We extracted DNA using DNeasy blood and tissue kit (QIAGEN) following manufacturer’s instructions. We amplified a 771 bp fragment corresponding to the PRNP open-reading frame using primers 223 (5’-ACACCCTCTTTATTTTGCAG-3’) and 224 (5’-AGAAGATAATGAAAACAGGAAG-3’), which do not amplify a known PRNP pseudogene [50]. PCRs were performed using QIAGEN Multiplex PCR Master Mix in a final volume of 10.00 µL: 5.00 µL 2x Master Mix, 1.00 µL 5x Q-Solution, 0.125 µL of 20 µM forward and reverse primers, 1.75 µL deionized H_2_O, 2.00 µL of 20 ng/µL DNA template. All PCRs were carried out with a program comprising 15 minutes of initial denaturation at 95°C, followed by 35 cycles (95°C for 30 seconds, 60°C for 90 seconds, and 72°C for 60 seconds), and final extension at 72°C for 10 minutes. Successful PCR amplifications were purified using a mix of 0.58 µL of Exonuclease 1 (20,000 units/mL; New England BioLabs), 0.58 µL of Shrimp Alkaline Phosphatase (1,000 units/mL; New England BioLabs), 8.84 µL deionized H_2_O, and 10 µL of PCR amplicon. We used the following thermocycler protocol for purification: 37°C for 30 minutes and 80°C for 15 minutes. Sanger sequencing was performed on an Applied Biosystems 3730 sequencing platform (Applied Biosystems) at the Penn State Genomics Core Facility, using both primers for each fragment.

We used Geneious v. 6.1.8 [51] to assess the quality of all forward and reverse sequences, keeping only those that had at least 75% of their bases with a Phred quality score of 20 (i.e. 99% accuracy in base calling) to avoid spurious results in base calling. We analysed sequence reads in R v. 4.1.0 [52] using the CWDPRNP package [53], which has been specifically designed to assess variability in the PRNP. This package is particularly useful for summarizing potentially polymorphic sites and creating primary and secondary base calls for each forward and reverse sequence to represent heterozygous genotypes. We aligned our sequences using the ClustalW algorithm implemented in the *Align* function, which also converts anti-sense reads to their reverse complementary sequence and trims them to a reference sequence. For the latter we used a 771 bp sequence of elk PRNP, which spans from start codon to stop codon (GeneBank accession number AF016228) [54]. We deposited one PRNP sequence per individual in GenBank (ON166945− ON167509).

We inferred haplotypes based on combinations of genotypes at different polymorphic sites using the program PHASE v. 2.1 [55,56]. We performed five independent runs to evaluate consistency in the results, setting the parameters as follows: 100,000 iterations, burn-in of 10,000 and drawing samples every 100 iterations [27,57] and default phase thresholds (90%). In a previous study of a white-tailed deer population in Arkansas, Chafin et al. [47] found 19 PRNP haplotypes based on 14 polymorphic sites. We decided to consider two of these sites to compare their haplotype frequencies with the ones we inferred: nucleotide 60 (similar position to the polymorphic site we found at nucleotide 63) and nucleotide 286 (linked to differences in susceptibility to CWD, equivalent to the polymorphic site at nucleotide 394 in elk that also encodes for reduced susceptibility).

We performed all the following analyses focusing only on the polymorphism at nucleotide 394 due to its effect on survival. We calculated deviations from genotypic proportions expected under Hardy-Weinberg equilibrium for each of our sampling years, using the HWE exact test implemented in the programme ARLEQUIN v. 3.1 [58] (1 million steps in Markov chain and 10,000 dememorization steps).

### Comparison to previous studies

We compared the genotypic frequencies obtained with those reported in other areas before CWD had been detected: three populations from Wyoming (JN = hunted, 55 samples collected near Jackson [11], WIND = hunted, 186 samples from Wyoming/Wind River Ranges, and ABSA = hunted, 148 samples from Absaroka Range [10]), one population from North Dakota (THRO = unhunted, 199 samples from Theodore Roosevelt National Park [10]), and one population from South Dakota (BH = hunted, 42 samples from the Black Hills region [11]). First, we used the *sampling* function in R base package to extract N genotypes from our dataset (in each case setting N to the sample size used for the population of reference) and calculated the frequency of each genotype in our subsample. We then applied the *replicate* function to repeat this process 1,000 times in order to obtain the average frequency of each genotype in our subsample. We pooled together the frequency of heterozygous (Met/Leu) and minor allele homozygous (Leu/Leu) under the category we called ‘less susceptible’, given that the frequency of homozygotes for the minor allele was <1% across populations [59]. We used Fisher’s exact tests for count data (α = 0.05) to compare the frequency of less susceptible genotypes between each of our simulated subsamples and the populations reported in previous studies.

### Distribution of less susceptible genotypes

In order to characterize the relative susceptibility of different hunt zones to CWD, we estimated the proportion of less susceptible individuals present in each zone. This could not be done for each of our sampling years separately due to some hunt zones being represented by a low number of samples in some years. Therefore, we decided to pool together our samples collected over the 7-year period 2014–2020. To assess the possibility of temporal changes in allele frequencies that could preclude us from combining different years, we first computed pairwise *F_ST_* comparisons between years [60,61] using ARLEQUIN v. 3.1 [58] (10,000 permutations). Even if the overall allele frequencies do not differ between years, that does not mean the distribution of less susceptible genotypes across hunt zones was the same over time. During our sample collection period two additional hunt zones were created, hunt zone 13 in 2015 and 14 in 2018. As a proxy to assess the stability of this spatial pattern, we divided our samples into three periods: 1) before hunt zone 13 was incorporated to the management area (i.e. samples collected in 2014), 2) after incorporation of hunt zone 13 but before incorporation of zone 14 (i.e. samples collected between 2015 and 2017), and 3) after incorporation of hunt zone 14 (i.e. samples collected after 2017). We performed Fisher’s exact tests for count data (Bonferroni corrected α) to conduct inter-period comparisons of the proportions of less susceptible elk, using only hunt zones that were represented by 15 or more individuals. These comparisons were as follows: zone 2 (period 1 vs 2, period 2 vs 3 and period 1 vs 3), zone 5 (period 1 vs 2), zone 6 (period 2 vs 3), zone 9 (period 2 vs 3), zone 10 (period 2 vs 3) and zone 12 (period 1 vs 2, period 2 vs 3 and period 1 vs 3).

We calculated Clark-Evans clustering coefficient [62] as a measure of spatial aggregation among all less susceptible individuals detected, using the *nni* function of the R package ´spatialEco´ [63]. This coefficient represents the ratio between mean observed nearest neighbour distance and mean expected nearest neighbour distance under the assumption of random distribution (values <1 indicate an aggregated distribution).

We divided the total number of our samples into hunt zones based on delineations corresponding to 2018–2020 (located within the Elk Management Area, Figure 1(b)) and mapped the proportions of heterozygotes using the inverse distance weighting (*IDW*) function, setting the power parameter to 3 and the cell size to 50x50m [59]. This function creates a raster object representing genotypic proportions as an interpolated surface. The seed points for this interpolation are the proportions of the different genotypes in each hunt zone, assigned to the centroid of that hunt zone. We repeated the process mapping the proportion of all less susceptible individuals (i.e. heterozygotes and minor homozygotes combined).
